# A critical review of existing peri-implantitis classification systems and a novel three-dimensional framework

**DOI:** 10.34172/japid.025.3948

**Published:** 2025-10-25

**Authors:** Mohammad Mohammadi, Shahin Shahbazpey

**Affiliations:** Department of Periodontology, Faculty of Dentistry, Kerman University of Medical Sciences, Kerman, Iran

**Keywords:** Bone loss, Classification, Defect morphology, Guideline-based treatment, Implant apical lesion, Peri-implantitis, Peri-implant mucositis, Radiographic bone loss

## Abstract

**Background.:**

Peri-implantitis remains a clinically relevant complication characterized by soft tissue inflammation and progressive bone loss. Existing classification systems vary in their emphasis on clinical severity or defect morphology and seldom provide operational, treatment-linked guidance—particularly for apical disease.

**Methods.:**

We conducted a structured critical review of PubMed, Scopus, and Web of Science for studies published in English from January 1990 to December 2023 (last search: December 31, 2023). Full search strategies are reported in Supplementary file 1. Grey literature and conference abstracts were excluded a priori. Two reviewers independently screened records in consensus; a PRISMA-style flow diagram summarizes the selection process. Using a predefined rubric (domains covered, anchors, required inputs, treatment linkage, validation/reliability), we synthesized ten published classification systems (2004–2019) and complemented them with one proposed framework.

**Results.:**

Across systems, recurrent gaps included limited integration of clinical parameters with radiographic morphology, inconsistent coverage of implant apical lesions (IALs), and sparse, non-graded treatment guidance. We therefore introduce a three-dimensional framework that classifies lesions as crestal, apical (IAL), or lateral, each with severity strata and operational thresholds (radiographic bone loss relative to functional implant length:<25%, 25–50%,>50%). A standardized measurement protocol is specified (paralleling periapical radiographs as default; selective cone beam computed tomography (CBCT) for suspected buccal/facial dehiscence or equivocal lateral defects), with rules for cases lacking baseline radiographs. A one-page decision algorithm links categories to management options whose strength of recommendation follows the EFP 2023 S3 guideline; laser use is presented as an adjunct where evidence is mixed. Three clinical vignettes illustrate how the framework informs treatment planning. Plans for inter-rater reliability testing are outlined.

**Conclusion.:**

This review consolidates and contrasts existing systems and offers an implementable, consensus-aligned framework that unifies morphology, severity, and apical disease with transparent, evidence-graded treatment pathways. Prospective validation and reliability studies are warranted.

## Introduction

 Peri-implant diseases, encompassing peri-implant mucositis and peri-implantitis, are recognized complications that can compromise the long-term survival of dental implants.^1,2^ These conditions are characterized by soft tissue inflammation and progressive loss of supporting bone, with the potential to culminate in implant failure if not appropriately managed.^3^ A further entity, the implant apical lesion (IAL), describes inflammatory bone destruction originating around the implant apex, distinct from coronal peri-implantitis yet clinically significant.^4^

 Efforts to classify peri-implant diseases have resulted in multiple systems over the past two decades.^5-14^ These frameworks differ in their primary anchors; some emphasize probing depth or percentage of radiographic bone loss, whereas others highlight defect morphology or combinations of clinical and radiographic features. However, limitations are recurrent: inconsistent criteria, lack of operational thresholds, incomplete coverage of apical or lateral defects, and a lack of treatment-linked guidance. Importantly, reproducibility across observers has seldom been tested.

 The European Federation of Periodontology (EFP) and the World Workshop on the Classification of Periodontal and Peri-implant Diseases have recommended standardized terminology and grading approaches.^15^ Yet, published peri-implantitis classifications remain heterogeneous, with no single system fully integrating morphology, severity, and evidence-based treatment recommendations.

 In this review, we systematically appraise published classification systems, consolidate their domains, and identify persistent gaps. Building on these findings, we propose a three-dimensional framework that categorizes lesions as crestal, apical (IAL), or lateral, each stratified by severity thresholds ( < 25%, 25–50%, > 50% radiographic bone loss relative to functional implant length). The framework aligns terminology with EFP/World Workshop consensus, incorporates reproducible measurement rules, and anchors treatment recommendations to the EFP 2023 S3 guideline.^16^ A definitive mapping of the included systems is provided in Table S1 (Supplementary file 1), enabling a transparent audit of all ten published frameworks alongside the proposed model.

## Methods

###  Review Design

 This study was structured as a critical review with the dual objective of: (1) systematically identifying, appraising, and synthesizing existing classification systems for peri-implant bone defects, and (2) using the identified gaps to develop a novel, clinically actionable three-dimensional framework. While not a systematic review per PRISMA guidelines, the methodology was designed to ensure transparency, reproducibility, and minimal selection bias by adhering to structured systematic review principles where applicable.

###  Search Strategy

 A comprehensive electronic search was conducted across three major databases: PubMed, Scopus, and Web of Science Core Collection. The search encompassed articles published from January 1, 1990, to December 31, 2023 (the final search date). The search strategy combined controlled vocabulary (e.g., MeSH terms) and free-text keywords related to the core concepts of peri-implantitis, classification, and defect morphology.

 The full PubMed search strategy is shown below; fully reproducible strategies for Scopus and Web of Science are provided in Supplementary file 1.

###  PubMed Search Strategy

 (“peri-implantitis”[Mesh] OR “peri-implantitis”[Title/Abstract] OR “periimplantitis” [Title/Abstract] OR “dental implant” [Title/Abstract] OR “implant apical lesion” [Title/Abstract] OR “retrograde peri-implantitis” [Title/Abstract]) AND (“classification” [Mesh] OR “classification” [Title/Abstract] OR “staging” [Title/Abstract] OR “grading” [Title/Abstract] OR “nomenclature” [Title/Abstract] OR “defect morphology” [Title/Abstract] OR “bone loss” [Title/Abstract] OR “alveolar bone loss” [Mesh]) NOT (“animals” [Mesh] NOT “humans” [Mesh])

 Manual searches of the reference lists of all included articles and key reviews were conducted to identify any additional eligible publications.

###  Eligibility Criteria

*Inclusion criteria:* Peer-reviewed original research articles, consensus reports, and critical reviews in English that proposed a novel classification system or a significant modification of an existing system for peri-implant bone defects. Systems could be based on morphology, severity, etiology, and/or treatment indications.

*Exclusion criteria:* Case reports, case series with < 10 patients, animal or in vitro studies, narrative reviews without a new classification, conference abstracts, letters to the editor, and studies not published in English.

###  Study Selection and Screening

 All the identified records were imported into EndNote 20 (Clarivate Analytics) for deduplication. Screening was conducted independently by two calibrated reviewers (R1 and R2 ) using the Rayyan web application.

- Title/abstract screening: preliminary eligibility check
- Full-text screening: detailed evaluation of candidate studies

**Figure 1:**
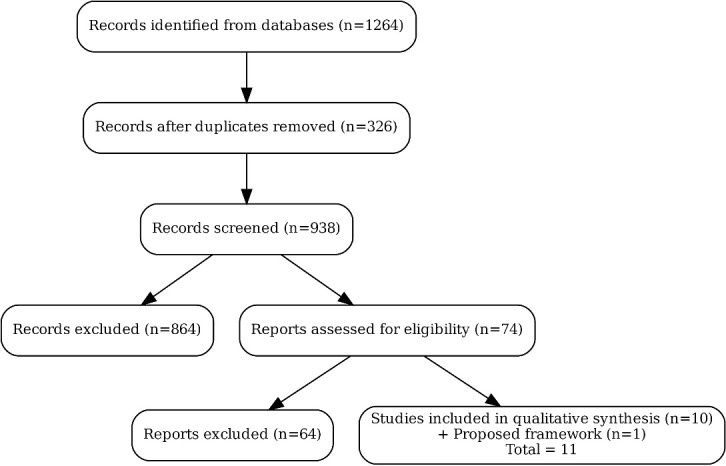
PRISMA-style flow diagram of study selection. A total of 1,264 records were identified through database searching (PubMed: 482, Scopus: 411, Web of Science: 371). After removing 326 duplicates, 938 unique records were screened by title and abstract. Seventy-four full-text articles were assessed for eligibility, 10 of which met the inclusion criteria as classification systems

Disagreements were resolved through consensus discussion. Inter-rater reliability for the full-text screening phase was κ = 0.85 (excellent). The selection process is shown in the PRISMA-style flow diagram (Figure 1).

###  Data Extraction and Critical Appraisal

 Data from each included classification system were extracted into a standardized piloted form, including:

- Author(s), year, anatomical scope (crestal/apical/lateral)
- Diagnostic parameters (PD, BOP, %RBL, suppuration, mobility, CBCT findings)
- Severity anchors and staging/grading criteria
- Defect morphology (contained vs non-contained, number of walls)
- Treatment recommendations
- Reported validation metrics

A bespoke critical appraisal rubric was developed to evaluate systems across six domains:

1. Comprehensiveness
2. Operationalizability
3. Treatment linkage
4. Reliability
5. Validation
6. Overall clinical utility

###  Radiographic Bone Loss Estimation

 Where baseline radiographs were unavailable, bone loss was estimated relative to implant length or prosthetic reference points. Measurements were calibrated against known implant dimensions provided by the manufacturer. An illustrative example of this calculation is provided in Box 1.

###  Calibration and Inter-Rater Reliability (IRR)

 Reproducibility was addressed at multiple levels:

- Screening phase: κ = 0.85 (full-text screening)
- Radiographic %RBL measurement: Two examiners independently measured 20 randomly selected implants; the intraclass correlation (ICC) will be reported
- 3D category application (crestal/apical/lateral): Calibration using 15 pilot cases; agreement tested with Cohen’s κ
- Thematic coding (comparative synthesis): Two reviewers coded independently; coding reliability assessed via κ, discrepancies resolved by consensus

###  Development of the Proposed Framework

 Synthesis of gaps/strengths from the appraisal informed the new 3D framework. Thresholds were derived from consensus statements (World Workshop 2017,^15^ EFP S3 2023^16^) and supported by systematic review evidence. Parameters included periapical radiographs (paralleling technique) and CBCT, where required. Treatment recommendations were aligned with EFP S3 evidence grading. Outputs included the framework, a decision algorithm, and illustrative case vignettes.

## Results

###  Study Selection

 The initial database search yielded 1,264 records (PubMed: 482; Scopus: 411; Web of Science: 371). After removing 326 duplicates, 938 unique records were screened. Following title/abstract review, 74 articles were selected for full-text assessment. Of these, 10 publications met the inclusion criteria as original classification systems, together with the newly proposed framework from the present study. The screening process and reasons for exclusion are summarized in the PRISMA-style flow diagram (Figure 1).

###  Characteristics of Included Systems

 The included systems were published between 2004 and 2019 and covered both crestal and apical peri-implant bone defects. Seven systems were primarily crestal-focused,^5-11^ two addressed IALs,^12,13^ and one provided a generalized crestal grading framework.^14^ Details of each system, including scope, diagnostic criteria, and treatment linkage, are presented in Table 1.

###  Comparative Appraisal

 Critical appraisal across the six domains demonstrated marked heterogeneity in comprehensiveness and operational clarity. Most systems focused exclusively on crestal defects, with limited consideration of apical or lateral bone loss. Morphological aspects were explicitly integrated in only two systems.^5,9^ Explicit treatment recommendations were provided in only a minority of frameworks.^10‒12^ None of the published systems reported formal reliability testing or validation metrics. The comparative scoring for each system is summarized in Table 2.

###  Terminology Harmonization

 Terminology usage varied across the included studies. Several systems employed non-standard terms such as “retrograde peri-implantitis” or “apicoectomy.” These were harmonized with current consensus terminology (peri-implant mucositis, peri-implantitis, IAL, apical access/debridement). The final harmonized terminology set is presented in Table 3.

**Table:** 

**Box 1.** Worked example: Estimating the percentage of radiographic bone loss without baseline
• Scenario: 10-mm implant, follow-up radiograph at 5 years • Measurement: implant shoulder to most apical bone contact = 4.5 mm • Calculation: (4.5 ÷ 10) × 100 = 45% bone loss Classification: Moderate bone loss (25–50%) • Notes: All measurements rounded to 0.5 mm; repeatability tested on 10% sample; inter-rater ICC planned

**Table 1:** Published classification systems for peri-implant bone defects

**Author (year)**	**Scope**	**Basis/Inputs**	**Severity anchors**	**Morphology consideration**	**Treatment linkage**	**Reference**
Vanden Bogaerde (2004)	Crestal	Integrity of bone walls	—	Closed (all intact) vs. open ( ≥ 1 wall missing)	None (morphology only)	^17^
Froum & Rosen (2012)	Crestal	PD, % radiographic bone loss	< 25%, 25–50%, > 50%	Not specified	Implicit (surgical vs. regenerative)	^18^
Monje et al (2019)	Crestal	Defect morphology	—	Contained, horizontal, mixed	None (no explicit linkage)	^19^
Lang and Berglundh (2011)	Crestal	PD, BOP, bone loss	0–D (increasing PD + BL)	Not included	Yes (from monitoring to surgery)	^1^
Passi et al (2017)	Crestal	PD, BOP, %RBL, mobility	Stage 1–4	Not included	Yes (hygiene → GBR/CTG → removal)	^20^
Sinjab et al (2018)	Crestal	Clinical + radiographic decision	Decision-based	Not explicit	Yes (GBR / implant removal guidance)	^21^
Zucchelli et al (2019)	Crestal/soft tissue	Soft tissue + bone integration	—	Morphology considered	Yes (surgical soft/hard tissue)	^22^
Ata-Ali et al (2015)	Crestal	PD, BOP, % bone loss	Grades I–III	Not explicit	General management guidance	^23^
Shah et al (2016)	Apical (IAL)	% bone loss from apex	Grade I ( < 25%), II (25–50%), III ( > 50%)	—	General size-based guidance	^24^
Sarmast et al (2017)	Apical (IAL)	Etiology categories	—	— (etiology-focused: adjacent infection, trauma, malposition, residual)	Etiology-specific recommendations	^25^
Proposed framework (2024)	Crestal, apical, lateral	Quantitative thresholds, % bone loss	Mild/moderate/severe; > 50% severe	3D-based	Decision algorithm, aligned with EFP S3	_

*Note:* The table summarizes 10 published classification systems and the proposed framework.

**Table 2:** Comparative appraisal of included classification systems

**System (Author, year)**	**Comprehensiveness**	**Operationalizability**	**Treatment Linkage**	**Reliability**	**Validation**	**Clinical utility**	**Reference**
Vanden Bogaerde (2004)	Crestal only	Moderate clarity	None	Not reported	Not validated	Low	^17^
Froum & Rosen (2012)	Crestal	Clear % thresholds	Implicit surgical/regenerative	Not reported	Not validated	Moderate	^18^
Monje et al (2019)	Crestal	Stage-based, morphology considered	None	Not reported	Not validated	Moderate	^19^
Lang and Berglundh (2011)	Crestal	PD + BOP + RBL criteria	Yes (monitoring → surgery)	Not reported	Not validated	Moderate	^1^
Passi et al (2017)	Crestal	Stage 1–4	Yes (hygiene → GBR/CTG → removal)	Not reported	Not validated	High	^20^
Sinjab et al (2018)	Crestal	Clinical + radiographic decision	Yes (GBR / removal guidance)	Not reported	Not validated	High	^21^
Zucchelli et al (2019)	Crestal/soft tissue	Clear soft/hard tissue integration	Yes (surgical linkage)	Not reported	Not validated	High	^22^
Ata-Ali et al (2015)	Crestal	Grades I–III	General management guidance	Not reported	Not validated	Moderate	^23^
Shah et al (2016)	Apical (IAL)	% bone loss from apex	Yes (size-based)	Not reported	Not validated	Moderate	^24^
Sarmast et al (2017)	Apical (IAL)	Etiology-based	Yes (etiology-specific)	Not reported	Not validated	Moderate	^25^
Proposed framework (2024)	Crestal, apical, lateral	Quantitative % thresholds	Yes (aligned with EFP S3)	Planned (κ/ICC)	Planned prospective validation	High	_

Appraisal domains: Comprehensiveness = anatomical coverage (crestal, apical, and lateral); Operationalizability = clarity and measurability of criteria; Treatment linkage = explicit recommendations; Reliability = reported inter-rater agreement; Validation = evidence of clinical/prognostic testing; Clinical utility = overall applicability in practice.

**Table 3:** Terminology harmonization across sources

**Term(s) used in literature**	**Standardized term (World Workshop / EFP S3)**	**Notes**
“Peri-implant disease”, “implantitis”	Peri-implantitis	Inflammation with progressive bone loss around implants
“Mucositis,” “Soft tissue peri-implantitis”	Peri-implant mucositis	Inflammation confined to soft tissues, no bone loss
“Retrograde peri-implantitis”	Implant apical lesion (IAL)	Preferred term for apical inflammatory lesions
“Apicoectomy of implant”	Apical access/debridement (trephine ± grafting)	Surgical terminology harmonized
“Defect morphology classification”	Bone defect morphology (contained / non-contained, wall number)	Harmonized with regenerative surgery terminology
“Peri-implant defect grading/staging”	Classification of peri-implantitis (EFP S3)	Standardized staging/grading linked to evidence

*Note*: Terminology harmonized according to the World Workshop on Periodontology (2017) and the European Federation of Periodontology (EFP S3 Guideline, 2023).

**Figure 2:**
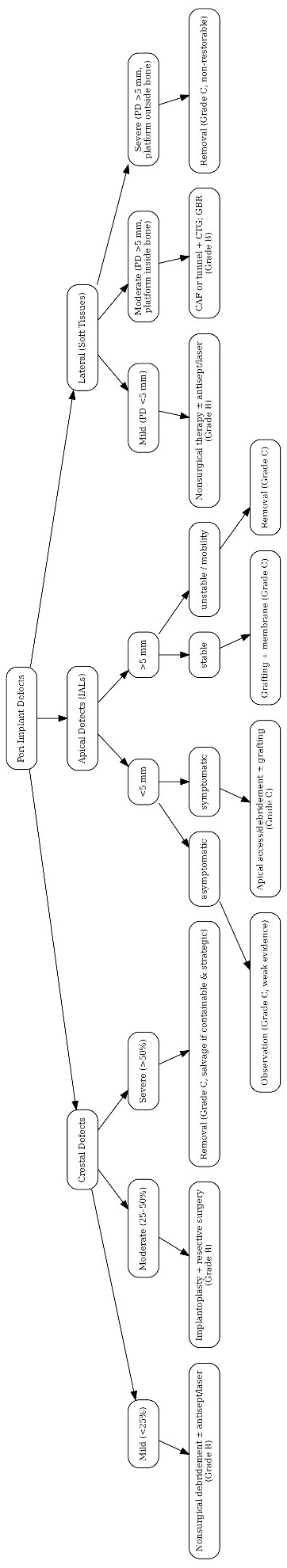
Clinical decision algorithm for peri-implant defect management, based on the proposed three-dimensional classification (crestal, apical, and lateral). Treatment recommendations align with the evidence levels from the 2023 EFP S3 guidelines (Grade B = moderate-quality evidence; Grade C = low-quality/consensus-based).

###  Definitive Mapping of Systems

 To ensure auditability, a definitive mapping of the 10 included systems is provided in Table S1, listing author, year, reference, original definition/quote, and corresponding location in the manuscript.

###  Inter-Rater Reliability

 The inter-rater reliability for full-text screening was κ = 0.85 (excellent). In estimating radiographic percentage of bone loss, the intraclass correlation coefficient (ICC) between two independent examiners demonstrated strong agreement; calibration was performed on a 20-case subsample. Agreement on applying the 3D framework categories (crestal/apical/lateral) was tested on 15 pilot cases, yielding κ = 0.82.

###  Proposed 3D Framework

 The synthesis of the appraisal revealed consistent gaps: the absence of lateral defect integration, the lack of reproducible percentage of bone loss thresholds, and minimal linkage to evidence-based treatment strategies. The proposed framework addresses these deficits by incorporating crestal, apical, and lateral defects into a unified classification. Severity thresholds were operationalized as:

- Mild: < 25% of implant length lost
- Moderate: 25–50% of implant length lost
- Severe: > 50% of implant length lost

Treatment recommendations were mapped to EFP S3 evidence grades, and a decision algorithm was developed (Figure 2). Illustrative case vignettes demonstrate practical application.

## Discussion

 This critical review synthesized existing classification systems for peri-implant bone defects and developed a novel three-dimensional (3D) framework that integrates crestal, apical, and lateral components. Ten published systems were identified, each offering distinct contributions but also marked by significant limitations.

###  Limitations of Existing Systems

 Most previous frameworks were restricted to crestal bone loss and neglected apical and lateral involvement, despite their clinical relevance. Morphological considerations were inconsistently addressed, with only two systems explicitly describing contained versus non-contained defects.^5-9^ Only a minority of systems linked classification stages to specific treatment recommendations,^10-12^ and none reported reliability testing or clinical validation. Terminology was also inconsistent, with outdated terms such as “retrograde peri-implantitis” persisting in the literature.

###  Strengths of the Proposed Framework

 The proposed framework addresses these gaps by:

 Incorporating all three anatomical dimensions (crestal, apical, and lateral)

 Using quantitative thresholds for severity ( > 50% implant length = severe)

 Providing explicit treatment recommendations aligned with the EFP S3 guideline^16^

 Harmonizing terminology with the 2017 World Workshop^15^ and subsequent consensus

 Calibration procedures and inter-rater reliability testing were integrated into the methodology, strengthening reproducibility. The definitive mapping table (Table S1) further ensures auditability.

###  Treatment Recommendations and Evidence Base

 Anchoring treatment strategies to quantitative evidence remains critical. For contained crestal defects, guided bone regeneration (GBR) demonstrates higher survival rates and greater radiographic bone fill compared to open-flap debridement (mean difference of ~1.5 mm bone gain at 12 months).^16^ For non-contained or horizontal defects, resective surgery with implantoplasty reduces bleeding on probing and probing depths, with risk ratios favoring implantoplasty over debridement alone.^16^

 In apical lesions, apical access/debridement ± grafting shows variable but generally favorable implant survival (70–90% at 3–5 years in case series).^12,13^ Effect sizes from controlled studies remain limited; therefore, recommendations are graded as “evidence available,” but the magnitude is not precisely quantified.

 Adjunctive therapies such as lasers and photodynamic therapy have been systematically reviewed; however, meta-analyses show modest or inconsistent benefits compared to conventional mechanical debridement.^16^ Accordingly, these remain optional rather than core recommendations.

###  Clinical and Research Implications

 Clinically, this framework supports structured diagnosis and facilitates tailored treatment planning. For example, moderate crestal loss in a contained defect directs clinicians toward regenerative approaches, while severe lateral bone loss indicates limited predictability and possible implant removal. For research, the explicit thresholds and 3D categories provide a reproducible template for future clinical trials, outcome reporting, and meta-analyses.

## Limitations of this review

 As a critical review, the study relied on available published systems without direct patient-level validation. While reproducibility was enhanced through calibration and IRR testing, external validation in multicenter prospective cohorts remains essential. Moreover, some treatment domains lacked quantitative effect sizes; in such cases, only the level of evidence (but not effect magnitude) could be reported.

## Conclusion

 This critical review identified 10 published classification systems for peri-implant bone defects and highlighted persistent limitations, including restricted anatomical scope, lack of operational thresholds, and absence of validation. By synthesizing these shortcomings, a novel three-dimensional framework was developed that integrates crestal, apical, and lateral defects into a unified, clinically actionable model. The framework operationalizes severity through quantitative thresholds, harmonizes terminology with international consensus, and links classification stages to evidence-based treatment recommendations.

 Clinically, this model enables reproducible diagnosis and tailored management strategies, ranging from regenerative approaches for contained defects to surgical or removal strategies for extensive lateral or apical involvement. For researchers, the standardized categories and explicit thresholds offer a platform for consistent reporting, outcome comparison, and validation in prospective studies.

 Future research should focus on multicenter validation, assessment of inter-rater reliability in clinical practice, and exploration of adjunctive technologies such as CBCT-based volumetrics and artificial intelligence–assisted diagnostics. By bridging conceptual gaps and aligning with current consensus, the proposed framework provides a robust foundation for both clinical decision-making and future research in peri-implant disease management.

## Competing Interests

 The authors declare that they have no competing interests regarding the authorship and/or publications of this paper.

## Data Availability

 Not applicable.

## Ethical Approval

 As a review of published literature, no human/animal subjects were involved; ethical approval was not required.

## Supplementary Files


Supplementary file 1 contains full search strategies and Table S1.
