# Supplementary material for: A critical review of existing peri-implantitis classification systems and a novel three-dimensional framework
Source: J Adv Periodontol Implant Dent. 2025 Oct 25;17(4):227–33. doi: 10.34172/japid.025.3948 (PMC12702084; doi:10.34172/japid.025.3948)
Supplement: Supplementary file 1 — contains full search strategies and Table S1. [file japid-17-227-s001.pdf]

Mohammadi and Shahbazpey, J Adv Periodontol Implant Dent. 2025;17(4):227-S1

doi: 10.34172/japid.025.3948

https://japid.tbzmed.ac.ir

Supplementary file 1

Full Search Strategies

Final search date: December 31, 2023

Scopus

(TITLE-ABS-KEY ("peri-implantitis" OR "periimplantitis" OR "implant apical lesion" OR "retrograde peri-implantitis"))
AND
(TITLE-ABS-KEY ("classification" OR "staging" OR "grading" OR "nomenclature" OR "defect morphology" OR "bone loss" OR "alveolar bone loss"))
AND
(LIMIT-TO(DOCTYPE, "ar"))
AND
(LIMIT-TO(LANGUAGE, "English"))
AND
(NOT TITLE-ABS-KEY ("animal" OR "in vitro"))

Web of Science Core Collection

TS=("peri-implantitis" OR "periimplantitis" OR "implant apical lesion" OR "retrograde peri-implantitis")
AND
TS=("classification" OR "staging" OR "grading" OR "nomenclature" OR "defect morphology" OR "bone loss" OR "alveolar bone loss")
NOT
TS=("animal" OR "in vitro")
Refined by: DOCUMENT TYPES: (Article) AND LANGUAGES: (English)

Notes: In all databases, a combination of controlled vocabulary (e.g., MeSH) and free-text terms was applied. Results were restricted to peer-reviewed articles in English, involving human subjects only, and limited to document type 'Article'. The final comprehensive search was executed on December 31, 2023.

Table S1. Definitive Mapping of Classification Systems

Author	Year	Reference	Exact quote/definition (≤25 words)	Location in manuscript
Bogaerde	2004	J Clin Periodontol. 2004;31:275–280.	“Closed defect = all bone walls intact; open defect = ≥1 wall missing.”	Table 1

Froum & Rosen	2012	Int J Periodontics Restorative Dent. 2012;32:605–613.	“<25% = Class I; 25–50% = Class II; >50% = Class III peri-implantitis.”	Table 1
Monje	2019	Clin Implant Dent Relat Res. 2019;21:681–691.	“Morphology types: contained, horizontal, mixed defects.”	Table 1
Lang	2011	J Clin Periodontol. 2011;38(Suppl 11):178–181.	“Staging A–D: PD + bleeding + bone loss thresholds.”	Table 1
Passi	2017	Clin Oral Implants Res. 2017;28:1292–1300.	“Stage 1–4 based on PD, bone loss, mobility.”	Table 1
Sinjab	2018	Int J Oral Maxillofac Implants. 2018;33:1137–1144.	“Decision tree integrating radiographic and clinical findings.”	Table 1
Zucchelli	2019	J Periodontol. 2019;90:1143–1153.	“Combined soft/hard tissue defects classification.”	Table 1
Ata-Ali	2015	J Clin Exp Dent. 2015;7:e377–e382.	“Grades I–III based on probing depth and % bone loss.”	Table 1
Shah	2016	Clin Implant Dent Relat Res. 2016;18:1047–1055.	“Grade I (<25%), II (25–50%), III (>50%) bone loss from apex.”	Table 1
Sarmast	2017	J Endod. 2017;43:1099–1104.	“Categories by etiology: adjacent infection, trauma, malposition, residual pathology.”	Table 1
Proposed framework	2024	This study	“Crestal/apical/lateral; severe ≥50% implant length lost.”	Table 1 + Figure 2
